# The impacts of implementing recovery innovations: a conceptual framework grounded in qualitative research

**DOI:** 10.1186/s13033-022-00559-2

**Published:** 2022-10-09

**Authors:** Myra Piat, Megan Wainwright, Marie-Pier Rivest, Eleni Sofouli, Tristan von Kirchenheim, Hélène Albert, Regina Casey, Lise Labonté, Joseph J. O’Rourke, Sébastien LeBlanc

**Affiliations:** 1 Department of Psychiatry, McGill University and Douglas Mental Health University Institute, 6875, Boul. LaSalle, Montréal, Québec H4H 1R3 Canada; 2 Department of Anthropology, Durham University, Dawson Building, South Road, Durham, DH1 3LE UK; 3 École de Travail Social, Université de Moncton, Local 386, 18, avenue Antonine-Maillet, Moncton, Nouveau-Brunswick E1A 3E9 Canada; 4 Department of Psychiatry, McGill University, Ludmer Research and Training Building, 1033 Avenue des Pins, Montréal, QC H3A 1A1 Canada; 5 Douglas Mental Health University Institute, 6875, Boul. LaSalle, Montréal, Québec H4H 1R3 Canada; 6 Ecole de Travail Social, Université de Moncton, Local 378, 18, avenue Antonine-Maillet, Moncton, Nouveau-Brunswick E1A 3E9 Canada; 7 Department of Occupational Science and Occupational Therapy, Faculty of Medicine, The University of British Columbia, 2211 Westbrook Mall T325, Vancouver, BC V6T 2B5 Canada

**Keywords:** Conceptual framework, Guidelines, Impact, Implementation science, Mental health recovery, Qualitative research, System transformation

## Abstract

**Background:**

Implementing mental health recovery into services is a policy priority in Canada and globally. To that end, a 5 year study was undertaken with seven organisations providing mental health and housing services to people living with a mental health challenge to implement guidelines for the transformation of services and systems towards a recovery-orientation. Multi-stakeholder implementation teams were established and a facilitated process guided teams to choosing and planning for the implementation of one recovery innovation. The recovery innovations chosen were hiring peer support workers, Wellness Recovery Action Planning (WRAP), a family support group, and staff recovery training.

**Methods:**

This study reports on data collected at the post-implementation stage. 90 service users, service providers, family members, managers, other actors and knowledge users participated in 41 group, individual or dyad semi-structured interviews. The interview guides included open-ended questions eliciting participants’ impressions regarding the impact of implementing the innovation on service users, service providers and organisations. We applied a collaborative qualitative content analysis approach in NVivo12 to coding and interpreting the data generated from these questions.

**Results:**

Eighteen impacts of implementing recovery innovations from the perspectives of diverse stakeholder groups were identified. Three impacts of working as an implementation team member and as part of a research project were also identified. Impacts were developed into a conceptual framework organised around four overall categories of impact: *Ways of being, Ways of interacting, Ways of thinking, and Ways of operating and doing business.*

**Conclusions:**

The IMpacts of Recovery Innovations (IMRI) framework version 1 can assist researchers, evaluators and decision-makers identify, explore and understand impact in the context of recovery innovations. The framework helps fill a gap in conceptualising service and organisation-level impacts. Future research is needed to validate the framework and map it to existing methods for studying impact.

**Supplementary Information:**

The online version contains supplementary material available at 10.1186/s13033-022-00559-2.

## Background

Mental health recovery is defined as “a way of living a satisfying, hopeful, and contributing life even within the limitations caused by illness” (p.527) [1]. There is a clear distinction in the recovery literature between “personal recovery” and “clinical recovery” [2]. The former relates to the definition above and to key pillars of the recovery process including: connectedness, hope, identity, meaning, and empowerment [2]. Reclaiming a meaningful life is a process based on self-determination and respect for the person as a valuable citizen of society [3]. Furthermore, the meaning of personal recovery continues to be explored in diverse settings and countries [4–6]. Clinical recovery is, on the other hand, tied to ideas of remission of symptoms, or return to a pre-illness state [7].

While clinical recovery and personal recovery are processes that can co-exist [8], clinical recovery paradigms tend to dominate traditional mental health services which can hinder personal recovery due to an overreliance on clinical, often coercive, interventions and prioritising professional experience over personal lived-experience and self-determined recovery goals [3, 9]. In contrast, recovery-oriented services support an individual's recovery journey by valuing professionals and service users equally, being person-centred and prioritising self-determination [3, 10, 11]. Since the 1990s, governments and mental health organisations internationally have embraced recovery as their primary paradigm for mental health services and guiding principle for mental health policy [3, 7, 8]. A recent systematic review of the implementation of recovery into services identified a diverse array of recovery innovations organisations can implement to work towards system-transformation [12].

### Implementing guidelines for recovery-oriented system transformation

An important element of transforming systems and services so that they align with the principles of personal recovery has been the publication of guidelines for recovery-oriented practice, such as those published in Canada in 2015 [13]. These guidelines include a chapter made-up of 4 sub-guidelines for transforming services and systems towards a recovery orientation (Chapter 6). These guidelines are a key resource to provincial and federal governments and organisations committed to implementing recovery into services; however, guidelines are notoriously difficult to implement [14]. In 2017 we received funding to implement Chapter 6 of the guidelines into seven mental health and housing organisations across Canada. We developed an implementation strategy combining external facilitation, the establishment of implementation teams and a 12-meeting planning process that guided implementation teams to take the guidelines and translate them into an implementation plan for one recovery-oriented innovation of their choice [15]. The strategy has since been developed into an online toolkit (www.walkthetalktoolkit.ca). Our research project outputs have so far included a study of the experiences of the implementation strategy [15], an evaluation of the CFIR Card Game we developed to translate implementation science for real-world use [16], and an analysis of the contextual factors that affected mid-implementation outcomes during the COVID-19 pandemic [17]. In this article we report on a conceptual framework for understanding the impact of recovery-oriented innovations grounded in an in-depth qualitative analysis of stakeholders’ perspectives.

### Distinction between impacts and outcomes

The literature in mental health recovery refers to both the outcomes and impacts of recovery-oriented innovations or interventions. While often the terms are used interchangeably [18], there is a tendency in the mental health recovery literature for qualitative studies to frame their studies in terms of impact [19–22], and for quantitative studies to frame theirs in terms of measurable outcomes [23, 24]. According to Belcher and Palenberg [25] who offer a rare in-depth analysis of the 2 terms, while both terms represent “effects” of an intervention, the lack of conceptual clarity around the distinctions and overlaps between the two is problematic. They recommend authors be as transparent in their definitions as possible, use meaningful qualifiers, and that impact always be defined from a system perspective, that is, not assume that the change seen can be fully attributed to the intervention alone. To be clear, we identify with Pace’s (1979) conceptual distinction between the 2 terms, as follows: The scope for impacts is broader than outcomes; impacts are grounded in subjectivity (feelings and experiences) rather than quantifiable “objective” effects; an evaluation focused on outcomes measures change, while an evaluation focused on impact will tell the story of the effect of change; and finally, outcomes tend to be shorter term effects, while impacts are longer-term effects [26]. The subjectivity, breadth and emphasis on story that qualify the concept of impact according to Pace [26] align well with the principles of recovery—for example recovery is individually-defined, a journey rather than a destination, and nonlinear. Also, outcome studies in recovery have tended to focus on personal-level outcomes, albeit with the inclusion of more subjective outcomes such as self-esteem, self-determination and empowerment [1]. Our focus here is on system transformation, so the broader concept of impact is a better fit for inductively investigating how impact is conceptualised at different levels and by different stakeholders. This includes but is not limited to impacts at a personal level. One of the aims of our research is to further clarify the concept of impact in mental health recovery through contributing a new conceptual framework based on how multiple stakeholders conceptualised impact at multiple levels and across a range of recovery innovations.

### Conceptual frameworks in mental health and recovery

Our goal in this research was to create a conceptual framework that answered our research question: What is the range of impacts experienced when implementing recovery innovations from stakeholders’ perspectives? The IMpacts of Recovery Innovations (IMRI) conceptual framework we present contributes to a growing body of conceptual frameworks in recovery research including the CHIME framework for personal recovery [2], the SPICE framework for service users’ and carers’ conceptualisations of recovery [27], a framework for recovery-oriented practice guidance [28], and a conceptual framework for characterising the impact of recovery narratives on recipients [19]. All of these are based on systematic literature reviews, a common approach to developing conceptual frameworks [29, 30]. To the best of our knowledge our conceptual framework on the impacts of recovery innovations is the first of its kind. It is unique in that it does not focus on the impact of one specific innovation, but rather on impacts common across a number of recovery innovations. Furthermore, the conceptual framework goes beyond the recipients of the innovation, including impacts for those indirectly targeted by the innovations, as well impacts on organisations and services as a whole. Following the presentation of our results and conceptual framework we compare the impacts we identified to existing research on the impact of recovery innovations, taking particular note of similarities, differences, and unique contributions.

## Methods

### Research settings and recovery innovations implemented

Seven organisations across 5 Canadian provinces participated. One organisation (New Brunswick 1) provided specialised mental health services, and 6 provided housing services to adults living with a mental health issue. 5 organisations were non-profits (New Brunswick 2, British Columbia, Manitoba 1, Manitoba 2, and Ontario), and the remaining two were publicly funded (Québec and New Brunswick 1). Ethics approval was obtained from 5 ethics research boards, as well as the board of directors of all participating organisations. All participants signed and were given a copy of the consent form. Service users were given a small monetary compensation for their time and travel expenses.

Each site created an implementation team that selected and implemented a recovery innovation that met their organisational needs and developed their own action plans and implementation plans [15]. Four types of recovery innovations were implemented (see Table 1). Sites that implemented peer workers as their chosen innovation hired one to two part-time peer workers to provide peer support to tenants living in supported housing. Sites that implemented a staff training programme in mental health recovery hired external trainers to provide a training programme to staff online or in-person. Two of the training programmes included some service users as participants in the training, and two adopted a train the trainer design. The site that implemented a family support group partnered with a national mental health organisation to provide a recurring 10 week group programme for family members. The site that implemented Wellness Recovery Action Planning (WRAP), a service user-led mental health self-management programme [31], contracted certified WRAP trainers to train WRAP facilitators within the organisation (both members of staff and service users). All but one site experienced delays or periods of postponement due to the COVID-19 pandemic [17], with only one site unable to resume the implementation of their innovation following the resignation of the hired peer support worker two weeks after the start of implementation (Manitoba 1). Due to the COVID-19 pandemic and related policies, some form of adaptation (for example switching to online formats) occurred in all sites [17].

**Table 1:** Study participants for post-implementation interviews per site & stakeholder group

Stakeholder group	Research sites and chosen recovery innovations							Total n = gender average age (SD)
	Québec (Peer workers)	Ontario (Staff recovery training)	Manitoba 1 (Peer workers)	Manitoba 2 (Staff recovery training)	New brunswick 2 (Staff recovery training)	New brunswick 1 (Family support group)	British columbia (WRAP)	
Tenants / service users^a^	6	1	0	2	1	2	4	16 (7F, 6 M,1 N-B, 2mis) 52.28 (16.06)
Family members^b^	1	0	0	0	0	1	0	2 (2F) 61 (1.41)
Service providers	4	8	2	5	4	10	5	38 (23F, 9 M, 1 N-B, 5mis 41.9 (12.42)
Managers	5	3	3	5	1	1	3	21 (12F, 7 M, 1 PNTA, 1mis 45 (9.03)
Housing proprietors	2	N/A	N/A	N/A	N/A	N/A	N/A	2 (1F, 1 M) 53.5 (10.60)
Knowledge users	1	1	0	1	0	1	0	4 (2F, 1 M, 1mis) 52.66 (11.71)
Other actors	2	1^c^	0	1	1^c^	2	1	7 (6F, 1 M) 47.71 (8.47)
Total	21	14	5	14	6	17	13	90

*WRAP* Wellness Recovery Action Planning, *N/A* not applicable (housing proprietors existed only in Québec), *SD* Standard deviation, *F* female, *M* male, *N-B* non-binary, *PNTA* prefer not to answer, *Mis* missing data (not answered by participant)

^a^Family members in New Brunswick 1 who attended the family support group service are referred to as service users

^b^Here family members refers to family members sitting on implementation teams

^c^The same trainer was hired in Ontario and New Brunswick 2 and was asked about each site in one interview

### Study participants and recruitment

90 people participated in the study including managers, service providers, service users, family members, knowledge users and other actors. Table 1 lists the number of participants by site and by stakeholder group along with gender and age characteristics. “Other actors” refers to newly hired peer workers (Québec, Manitoba 1), external and internal WRAP trainers (British Columbia), support group facilitators (New Brunswick 1), and externally contracted recovery trainers (Ontario, Manitoba 2, New Brunswick 2). Twenty-six of the 90 participants were implementation team members and participated in more than one data collection activity to explore their dual roles; for example, a manager may have participated in the Manager focus group, and the implementation team focus group. The stakeholder group “housing proprietors” was specific to the Québec site. These were the owners of the supported housing accommodation contracted by the public health mental health hospital. Sampling was purposive. All implementation team members were invited to participate, and individuals with varying levels of exposure to the innovation were recruited for each key stakeholder group beyond the implementation team. Due to the COVID-19 pandemic, policies restricting in-person gatherings, and poor access to adequate internet and IT devices, in some sites it was not possible to recruit the intended sample of service users. There were specific recruitment challenges in New Brunswick 2 due to the pandemic and to relocation of tenants to a new building underway in the organisation. Also related to the pandemic context was the unforeseen resignation of the newly hired peer worker in Manitoba 1 only 2 weeks after being hired. Because this meant some stakeholder groups had no or extremely little exposure to the innovation, recruitment focused on those groups involved in planning.

### Data collection and data management

Forty-one semi-structured interviews were conducted: 15 individual, 15 group, and 11 dyad interviews. Group interviews included three to nine participants. 27 were conducted in English, and 14 in French. All but one interview occurred between November and December 2020. The 41st interview was conducted in June 2021 due to difficulties contacting a research participant. Implementation began approximately 8 to 13 months before post-implementation data collection except for in one site where implementation had started 2 months prior to post-implementation data collection (New Brunswick 2). Interviews lasted on average 60–70 min, the shortest being forty minutes and the longest 1 hour and forty minutes. Due to COVID-19 restrictions, 39 interviews were conducted online. Senior researchers (MP, M-PR, HA, RC) conducted 28 interviews, and 13 interviews were conducted either by senior researchers and research assistants together, or research assistants on their own.

The interview guide had 2 sections, one that explored participants’ perspectives on the impact of the innovations, and the other that explored implementation factors. In this article, we report only on the analysis of the impact of the innovation questions. The same interview guide was used for all participants (see Table 2), except for one site (Manitoba 1) where we used a modified version because the innovation was implemented for a very short amount of time. Although questions targeted the innovations' impact, implementation team members also spoke to the impact of the research project or participating in an implementation team. All interviews were audio/video-recorded and transcribed verbatim. Transcription accuracy was checked against the recording by a member of the research team for 5 interviews. Interviewer reflection diaries were completed for 30 interviews. Interview transcripts and reflection diaries were imported into NVivo12 [32]. Two researchers (MW, TvK) developed a system for labelling the data [33] within NVivo12. We used Case Nodes [32] to code each transcript to three levels of cases: innovation, site, and stakeholder group. Memo links [32] were added to all transcripts, process log memos were created for each researcher to record their work in, and finally an analysis memo created for each stage of the analysis to record key decisions and notes from data analysis meetings.

**Table 2:** Interview guide questions on impact

1.What has been the impact of implementing [name of the innovation] on service users/tenants?
2.What has been the impact of implementing [name of innovation] on service providers?
3.What has been the impact of implementing [name of innovation] on you individually as a [service provider/service user/manager/knowledge user/trainer]?
4.From your point of view, what has implementation of [name of innovation] achieved in the organisation?
5.[To implementation team members, and managers and housing proprietors] Has participating in this project triggered any other recovery-oriented changes in the organisation? For example, a new service, a new policy, changes to everyday practices?

### Data analysis

We employed Forman and Damschroder’s approach to qualitative content analysis, which they describe as “a generic form of data analysis…comprised of an atheoretical set of techniques” (p. 40) [33]. It is an approach well suited to answer practical implementation questions by focusing on the informational content of the data [33]. While the concept of an atheoretical approach is a source of debate among some qualitative researchers, it has wide acceptance in the health sciences [33]. Using a collaborative approach to analysis [34] 5 researchers (MP, MW, M-PR, ES, TvK) met 31 times as a group over 9 months to complete the qualitative content analysis in 3 phases: immersion, reduction, and interpretation [33].

### Stage 1, immersion: engagement with the data

During immersion, researchers become acquainted with the data before arranging it into smaller units for analysis, which we accomplished by following several techniques laid out by Forman and Damschroder [33]. One researcher (TvK) read all 27 English interview transcripts as well as reflection diaries which interviewers completed after conducting their interviews. Three other researchers (MP, MW, M-PR) divided and read the remaining 14 French interview transcripts. Each researcher re-read and memoed their assigned transcripts, writing notes that explored key ideas, impacts, and emerging patterns.

### Stage 2, reduction: developing a consistent approach to the data

In this stage of analysis our focus was on developing a systematic approach to data reduction by accomplishing three goals: “(1) reduce the amount of raw data to that which is relevant to answering the research question(s); (2) break the data (both transcripts and memos) into more manageable themes and thematic segments; and (3) reorganise the data into categories in a way that addresses the research question(s)” (p. 48) [33]. We realised these goals by first developing a code book and then coming to an agreement on coding.

As our goal was to create a conceptual framework, we developed our initial code book solely through inductive analysis. We drew on our memos to each draft an initial list of impacts (codeswe saw being described in the transcripts. Following this we used Ideaboardz [35]—an online app for creating “sticky notes” that can be pasted on a virtual board, moved around and merged. Four researchers (MP, MW, TVK, M-PR) were assigned a column to post their sticky notes in (one sticky note for one impact). A fifth researcher (ES) looked for similarities across the sticky notes in real-time and began drafting a list of common impacts, which was then discussed and consolidated by the team into 29 draft impacts which served as our initial list of codes. In line with qualitative content analysis, 2 researchers (MW and TvK) worked on naming and writing definitions for each draft impacts and entered them into NVivo12 as our initial coding book.

In order to attain coding agreement, four transcripts were double coded by 2 researchers (MP, MW, ES, TvK). Coding comparison was undertaken in NVivo12 to refine codes. We used an iterative approach and met eleven times during the coding process to compare coding, review, merge and refine codes and their definitions, at which point we were satisfied we had attained coding reliability. One researcher (TvK) coded the remaining English transcripts, and three other researchers (MP, MW, M-PR) coded the remaining French transcripts.

### Stage 3, interpretation

At this stage the coded data underwent further analysis, interpretation and synthesis in order to generate the final results [33]. We began by undertaking a stage of reviewing codes described in thematic analysis [36]. This involved 2 authors reading all of the data extracts by code impact to ascertain whether the data fit meaningfully together or whether any re-coding was needed. Our approach to developing “code reports” (p. 57) [33] in the process was the following: 2 authors read the data for each code and completed a form with the following prompts (1) impression of the similarity between data, (2) whether change was needed to the name or definition of the code, (3) whether any data needed to be re-coded elsewhere, (4) what if any sub-coding, merging or splitting of codes may be needed and (5) anything surprising in the data. Over the course of 13 meetings, we compared our code reports and merged and re-coded data as needed to arrive at a final list of codes.

We then drafted Concept Maps in NVivo12 with our final list of codes and over the course of 7 meetings worked together to visually group those that we saw as related together under overall categories of impact (see Additional file 1). These Concept Maps were consolidated into our final conceptual framework. In order to check whether any of the categories of impact solely represented the experience of one site, innovation type or stakeholder group, we ran Matrix Coding Queries in NVivo12. Our conclusion was that all four categories of impact were well supported by the data. All contained data from all sites, innovation types and stakeholder groups except for *Ways of interacting* which did not have any data extracts from housing proprietors (though this was a very small stakeholder group present in only 1 of the 7 sites). Finally, we sub-coded all data for each impact into “impact of the innovation” and “impact of project, research or implementation team” in order to identify whether any impacts were exclusively related to the implementation process rather than the specific innovation. Three such impacts were uncovered and are represented in the conceptual framework separately.

## Findings

**Fig. 1:**
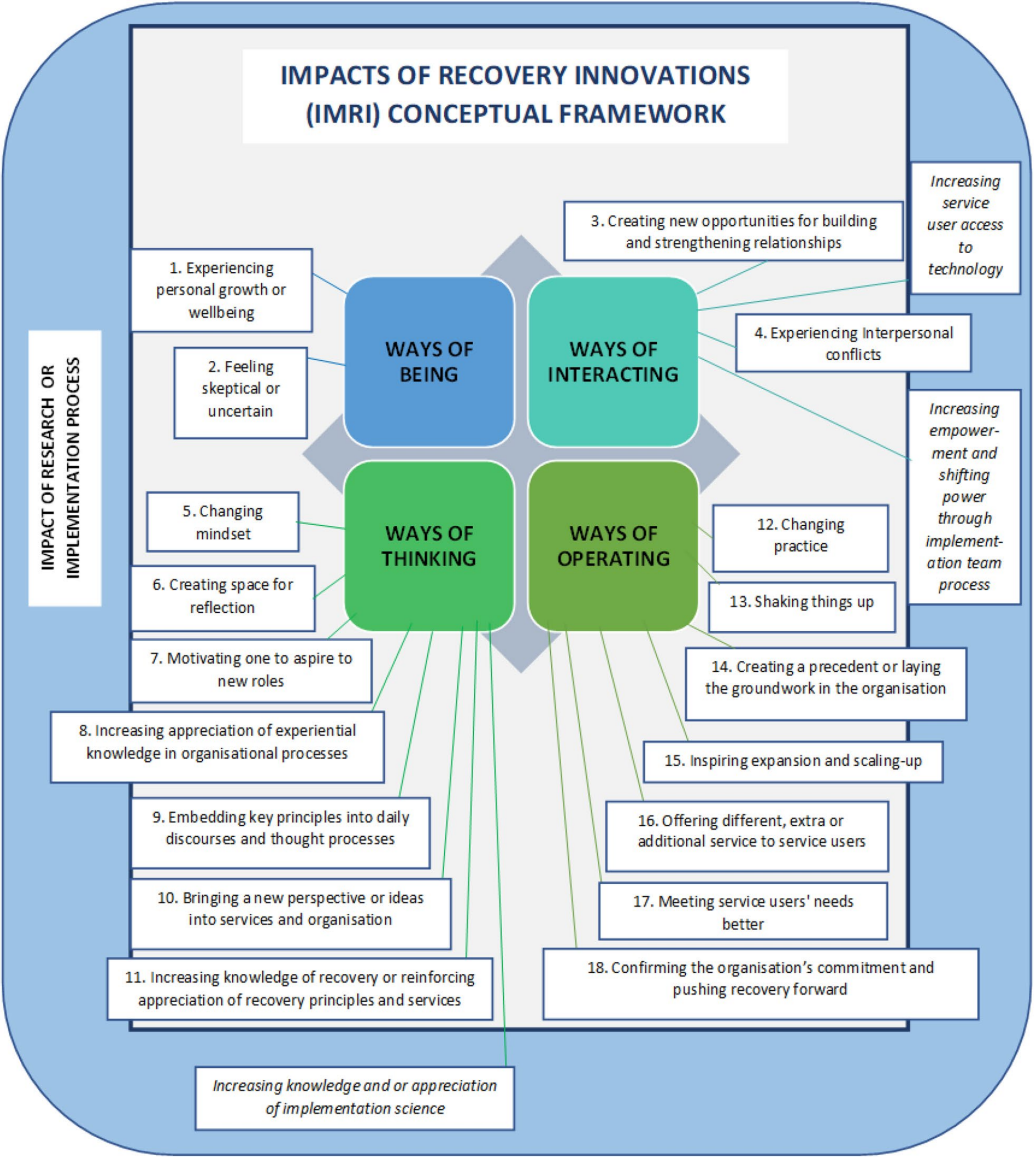
Conceptual framework of IMpacts of Recovery Innovations (IMRI)

Our analysis and interpretation of the data culminated in identifying 18 impacts of implementing recovery innovations from the perspectives of diverse stakeholder groups. In our conceptual framework (Fig. 1), we grouped these 18 impacts conceptually into four overall categories of impact: ways of being, ways of interacting, ways of thinking, and ways of operating and doing business. Below we present our findings by category of impact (see Additional File 1 for Concept Maps which guided our write-up of the findings). Quotes from interviews in French were translated by authors into English. Quotes are identified by stakeholder group of the respondent and the innovation implemented at their site. Self-reported sociodemo-graphic factors such as participants’ gender, age, and cultural background were not taken into account in data analysis; our primary focus was on understanding and revealing participants’ perspectives based on their stakeholder group.

Impacts that fall completely within the grey rectangle contain data exclusively relating to the implementation of the selected recovery innovation. Impacts that fall completely within the blue rectangle with rounded corners contain data exclusively relating to the implementation process. Impacts overlapping the grey and blue rectangles contain data predominantly relating to the implementation of the selected innovations, but also some data segments related to implementation process.

We noted that participants occasionally not only spoke about the impact of implementing the recovery innovation but also the impact of the implementation or research process itself, that is, the experience of using implementation teams and having external facilitation from a research team. In one interview with a site that had to stop implementation of their innovation after only 2 weeks, we explicitly targeted interviewees’ perception of the impact of the process as a whole. We paid attention to this in all of the data by systematically identifying whether participants were talking about either the innovation, or the process. In Fig. 1, impacts that fall completely within the grey rectangle contain data exclusively relating to the implementation of the selected recovery innovation. Impacts that fall completely within the blue rectangle with rounded corners contain data exclusively relating to the implementation or research process, of which there were three impacts. And finally, impacts overlapping the grey and blue rectangles contain data predominantly relating to the implementation of the selected innovations, but also some data related to the implementation or research process. We provide our interpretation of this in the discussion section.

### Ways of being

Ways of being is a category of impact that relates to how individuals can experience transformation at a personal level as a result of implementing a recovery-oriented innovation. In this case, this impact related predominantly to a sense of personal growth or wellbeing, either of service users, or of service providers (Fig. 1). This was especially the case, though not exclusively, in the sites that implemented innovations targeted towards service users, such as WRAP, peer support workers, and a family support group. Multiple stakeholder groups noted that one impact on service users they observed was a “coming out of one’s shell” (Service provider, peer worker innovation) that took the form of service users being better able to express their own needs, desires and emotions. A tenant in one of the sites that implemented peer support mentioned *“ I see that some other residents in my house are basically um, like, they are able to discuss like how they feel and things like that. So, they can do it properly and all that kind of stuff. So, they’re able to do that. And also they have one on one conversations as well so it’s, it’s really good.* Learning was also an important element of personal growth, either learning something about oneself and how to care for oneself, or learning specific tools that can be applied in everyday life. Three tenants who attended WRAP specified:

“…it’s helped me in identifying triggers, identifying when I go into crisis… I learned how to utilize coping skills” (tenant 1); “And the WRAP plan really helped me out. I was able to go to the hospital without the emergency line and able to get fixed up and taken care of without having to explain a lot….

I learned how to form a crisis plan” (tenant 2)”; “Yes, I’ve actually used the program in regards to listing and recognizing days when I’m better and days when I’m not, and days when I’m not, I know who I can reach out to in terms of my list, in terms of my step of things that I need to do. I’ve used that thing multiple times, multiple times. Oh yes, it’s great! It’s absolutely fabulous (tenant 3).

Personal growth was also characterised by an overall more positive outlook on life, increased self-esteem and confidence, emotional improvement in the sense of feeling happier, more positive, more hopeful, more relaxed and less stressed, and feeling stimulated in one’s life. A service provider described the impact on service users as follows:

*I suppose I’ve seen in the last year, even in the last 6 months, a tremendous growth in their openness and willingness to share and engage with us. When I first came here, they just wouldn’t, they didn’t speak to me for a long time, and now they come in, pop in the door, engage me. (service provider, staff training innovation)*

As for the personal growth of service providers, this took the form of staff having a sense of accomplishment, experiencing a personal sense of awakening and increased confidence in one’s work.

*So, I would add that it’s an opportunity to learn and grow, right? Because learning is always fluid like you may, we can’t learn enough because from out of the blue, somebody may have a different perspective or deeper insight or whatever, so for me the impact of this for both service providers and service users, would be the opportunity for more growth and development. (Service provider, staff training innovation)*

For a minority of service users and staff the implementation of a recovery innovation brought on *a way of being* that was hesitant about or resistant to change, particularly at the start of implementation. A manager, speaking of the impact on staff, described the following:

I think at the beginning they were a bit reluctant, they don’t necessarily have the time, the resources. There were some sticks in the wheels along the way, well not sticks, but, you know, the.

staff are so busy, so to add one more programme, one more task, they were hesitant. (Manager, family support group innovation)

### Ways of interacting

The implementation of recovery innovations also impacted the way people interacted with one another, as did the implementation process itself. In the case of the impact of the recovery innovations, in the vast majority of cases, these were positive impacts such as new opportunities for building and strengthening relationships. These took the form of an improved sense of community and inclusivity, staff working together and supporting each other, improved interpersonal relationships in personal and home life, improved or increased connections between people and different service points, improved cohesion among service users and staff, and building relationships with external organisations and partners. For example, at the Québec site where peer support workers were implemented into housing services, multiple stakeholders noted how the group peer worker sessions helped to build relationships between tenants in positive ways. A tenant on the implementation team described how they learned about each other and could relate to one another in new ways.

He [fellow tenant] talks about his boats, where he travelled and everything. Everybody has a past here, not just the mental grounds, but everybody has a past, they had a family, they lived somewhere before, they travelled somewhere, you know? They are all different, but we all relate with each other. (Service user, peer worker innovation).

Another service user at the British Columbia site who participated in the WRAP programme being implemented highlighted the impact on the way they, as a service user, related to staff:

*I would like to say that because of WRAP, and again just in my own personal demeanour and how I carry myself, that the interactions that I do have with the staff are just on a better level. (Service user, WRAP innovation)*

Similarly, in the Manitoba 2 site that implemented a staff recovery training programme that was open for some tenants to participate in, a manager expressed how much they valued the opportunity to build relationships with service users:

*And I really would also like to say that I value and honour having deeper relationships with some people that were part of the training, and that includes [service user’s name] and [service user’s name], and then maybe some other areas that I don’t normally get a chance to work with, other staff. (Manager, staff training innovation)*

The implementation process, particularly the implementation team process, was also referred to as having impacted interactions among stakeholders by empowering members and shifting power. In particular, the implementation team process empowered stakeholders not typically at the decision-making table, including service users, family members, even staff, to participate. It was seen as a bottom-up approach, one where there was a sharing of power for decision-making, and one that could increase members’ confidence and pride in their work. The implementation team process also impacted service users by increasing their access to technology since participating organisations were asked to ensure access to a laptop, tablet or smartphone for service user members of the implementation team to enable their full participation. This need was reinforced when the COVID-19 pandemic pushed in-person meetings online.

Although the impact on ways of interacting were predominantly positive, there were also some examples given of increased interpersonal conflict, usually as a result of norms or power relations being questioned in the recovery-transformation process (see Ways of operating and doing business).

### Ways of thinking

The recovery innovations led to shifts in mindset, that is, a change in thinking from what one thought in the past to a new way of thinking that was more recovery-oriented. Examples of this included housing proprietors in Québec changing their preconceived ideas around which tenants might engage with peer support workers. Similarly, but from a tenant’s perspective who participated in WRAP, just offering WRAP in the building and seeing some benefit made other tenants think differently. For family members participating in the support group, a key change in mindset was to stop blaming oneself and feeling guilty. A service provider who participated in recovery training described the following mindset change:

*Well, I think I’m intentionally more aware of it, I notice it in what we’re doing but also what’s happening in our community, what our residents are doing, and I’m sure that some of this happened before but I never had the sort of mindset to catch that, capture that and focus on it and then being able to build on that. (Service provider, staff training innovation)*

Similarly, another service provider from a different site who participated in a recovery training said:

*I would say the biggest impact for me is just like mindset, like the way that you think of the situation or the way that you approach the situation and like I find myself kind of like challenging like old beliefs or old like things that when I started working here were just like a trickledown effect from people previous to me, but now I think of things a lot differently, like I don’t just do things because that’s the way it was taught to me or that’s the way people did it for 20-30 years. (Service provider, staff training innovation)*

The recovery innovations were reported to have increased peoples’ knowledge of recovery but also reinforced appreciation of recovery and its principles, including the inclusion of experiential knowledge in organisational processes. In a direct way, the implementation of recovery innovations brought a new perspective or set of ideas into services for people to think about. For example, in all three sites that implemented staff recovery training, participants described one impact as being the inclusion of service users in hiring processes for new staff, as well as other kinds of decision-making committees. The introduction of new ideas not only changed individuals’ ways of thinking, but also led to an exchange of ideas formally and informally in the organisation which pointed to a subtle shift towards embedding key principles of recovery into everyday conversations and processes. Just creating space for reflection on what was learned was itself an impact, as described by the following manager:

*…having an opportunity to sort of critically evaluate what we’re doing and whether we’re actually doing it very well or aligned with what we now know recovery means. (Manager, staff training innovation).*

Innovations also had the impact of motivating some individuals to aspire to take on new roles representing a new way of thinking about themselves and also a change in how others thought about them. This often referred to service users expressing an interest in taking on a new role, for example becoming a peer worker themselves, becoming involved in staff training or joining committees. It was clear that as a result of the implementation of recovery innovations, new spaces and opportunities for reflection and for learning were created and, in many cases, had a profound impact on collective and individual thinking about recovery and the services provided to people living with a mental health challenge.

Finally, an important change was that stakeholders, particularly managers and staff on the implementation team, gained increased appreciation and knowledge of implementation science, in particular the importance of planning and engagement.

*For me personally it has provided me an awareness for the process that is involved in implementing a new innovation within a community setting. The steps involved, the complexity, all the forethought needed prior to an innovation and the process of even implementing and getting buy-in from stakeholders, it kind of opened my eyes to that personally. (Service provider, peer worker innovation)*

### Ways of operating and doing business

The implementation of recovery innovations impacted how organisations and staff within them operated. In a direct way, one of the impacts was simply having an additional service, programme or option to offer service users in contexts where programming can sometimes be lacking. Recently trained WRAP facilitators framed it as giving them “an extra tool that we didn’t have before” (Service provider, WRAP innovation). More profound than just having something else to offer service users, was that organisations were meeting the needs of service users better as a result of the recovery innovation, in particular by making service users feel more supported, and going beyond what traditional services could offer by drawing on the power of lived-experience rather than professional expertise. This was the case for innovations that were directed specifically to tenants, rather than staff training. For example, in the case of peer worker innovations, a manager put it as follows:

*From what’s been reported to me, it’s very beneficial. I think service users feel accompanied by people who are their equals, they speak the same language, who also come at things from a different angle or another way with them, grounded in the day-to-day, in accompaniment, they will take the time to touch on different aspects with them. (Manager, peer worker innovation)*

A tenant reflected especially on how transparent the peer worker was about her own experience with psychiatric hospitals and that this really surprised them.

I found it really fantastic to have someone so transparent in my life, and I was very transparent with her too, and I feel that it helps, it helps more than a psychologist or psychiatrist. (Service user, peer worker innovation).

For a service provider, offering WRAP to service users had the perceived impact of showing service users “we do care and then we are supportive, and we are there for them.” (Service provider, WRAP innovation).

Feeling supported was also a clear impact of the family support group innovation. The lived experience of one of the facilitators was also noted positively:

I really liked how it was really open, I liked it when they [facilitators] shared personal things in their life, like it showed us that they are not above us, like we are all together in the same boat, and I found that a really nice way, a nice collective approach I guess you could say. (service user, family support group innovation).

The recovery innovations had an impact on how individual staff members operated in their roles, in that they changed some of their practices. For both Manitoba 2 and New Brunswick 2 that implemented a staff training programme, one change in practice was to be less punitive when it came to dealing with “rule-breaking” in their housing service.

*[before] There were like clear boundaries around, you know, somebody screams at the staff, they’re out for 3 days, you know? And that’s really shifted and changed, where we are speaking to the person, having conversations about what’s going on and you know? So, talking about the behaviour rather than you know drawing a line in the sand and saying this is what you can do, and this is what you can’t, so a little bit of self-determination starting to be more normal than, than not, and I think that’s been like evident like since the beginning of the training. (Manager, staff training innovation)*

Even in sites where staff were not the immediate focus of the innovation, staff reported changes in practice as a result of the recovery innovations. In Québec a service provider described:

I have seen how their [peer workers’] interactions have also helped me work better and know the better approach to work with them [service users] because of what the peer advocate has been able to achieve with them. (Service provider, peer worker innovation).

By changing old ways of doing things, implementation of recovery innovations also had the impact of “shaking things up” in the organisation. This implied changes that disrupted norms and power dynamics and that could, at times come at a cost for some. The most extreme example of this was in one site that implemented staff training. The training challenged the organisation to look at its ways of doing things and as a result some staff members took-on advocating for change to key organisational documents including the organization’s mission statement, in an effort to use “a more inclusive and positive language.”. This led to a termination of employment of one staff member and a suspension of two others as the Executive Director felt that they “were trying to eliminate the core mission of the organization. Following “a formal grievance” against the director, the latter resigned. One of the staff persons who was temporarily suspended and described the previous six months as “a nightmare”, reflected:

And so, I mean if this recovery piece—you know, project—never came about we probably wouldn’t have gotten to this sort of, this big bang [laughs] and explosion and that’s where I mean the challenges—you know, the benefits of all this really outweigh [the challenges] and if I look back on things, I probably would not have it done it any other way. (service provider, staff training innovation).

At the organisational level both participation in a research project and the implementation of recovery innovations effectively confirmed organisations’ commitment to recovery and pushed recovery forward. Many of the organisations already had standing internal and external mandates to implement recovery into services, and therefore implementation of the innovations aligned with their goals. In the process of implementing the recovery innovation, the commitment of the organisation, staff and senior management was reinforced. It also created a precedent and laid foundations in the organisation. For example, laying the foundations in human resources departments for hiring a peer worker, establishing a pay scale, and making temporary contracts permanent. The success of the innovations gave those involved confidence that they had succeeded and could succeed again. In fact, another impact observed in all sites was that stakeholders were inspired to expand or scale-up their innovations. All described wanting the innovation to grow in the organisation and plans for achieving the growth. The family support group for example, went online due to COVID-19 and was expanded to participants across the county and plans were being made to offer it province-wide. A service provider’s enthusiasm is evident in the following statement:

*So me, I’m super motivated about this project. Whatever amount of energy it might take in terms of the organisation, in terms of time, in terms of meetings, it is worth it if the result is getting things into action, and that’s what we are doing. So, from my side I say, we’ve got the support of managers, we’ve got the support of the director. What more can you want? We had everything in place to be able to make it work and I think it will continue, it will continue to grow, it will continue to take-up its place and that’s fantastic. (service provider, family support group innovation).*

Thus, from the perspectives of stakeholders, the implementation of recovery innovations, whether it be a peer support worker, WRAP, a family support group or staff recovery training, had profound and multifaceted impacts on ways of operating and doing business.

## Discussion

To the best of our knowledge, we have developed the first conceptual framework of the impacts of implementing recovery innovations. The framework is grounded in primary qualitative data reflecting how stakeholders conceptualise impact. The research from which we developed the framework was innovative in 2 ways. Firstly, in our study, participating organisations were not told what to implement. Rather, the researchers facilitated a process where implementation teams chose a recovery innovation and planned for its implementation [15]. As a result, our study does not focus solely on one innovation, but on four recovery innovations implemented across seven participating organisations. Despite this diversity, we found that similar impacts were mentioned across organisations and innovations. We therefore believe the conceptual framework has merit for thinking about the impact of implementing recovery innovations in general, regardless of the exact recovery innovation being implemented. The fact that participants also highlighted the impact of the process, or their involvement in the research and implementation teams, reflects how the “process was itself a form of transformation” (p. 1) [15]. Therefore, the process should not be overlooked. An implementation process that integrates recovery principles such as choice and empowerment [2, 11]] and co-production approaches [37], can enhance and reinforce the impact of implementing recovery innovations. The fact that many impacts we identified were also supported by data about the process, supports this argument.

Secondly, our approach was novel because participants included multiple stakeholders who were asked to reflect not only on the impact of the innovations on those directly targeted such as service users or service providers, but on impacts on the organisation they were implemented in and those who were not direct recipients. Research to date has predominantly focused on the impact of the innovation on individuals receiving the innovation [38, 39]. For example, looking at the impact of staff training on the staff receiving training [40, 41], or studying the impact of a new recovery-oriented service for service users on service users themselves [42]. Crowther and colleagues’ study on the impacts of a recovery college is a notable exception as they go beyond the impact on students and investigate impact at other levels, including staff level (managers, administrators, trainers), service level and societal level [38]. Our approach enabled us to move beyond personal-level impacts and explore organisational-level impacts, but fell short of explicitly investigating societal level impacts. However, since a core aim of implementing recovery is to transform services and systems, ways of conceptualising impact at the service user, service provider and organisation level are needed. Potential harms are also infrequently studied or reported [38] and we do include these in our framework. However, as others have noted, it is not always easy to simply categorise an impact as positive/helpful or negative/harmful as it depends also on contextual factors [19]. What might be a negative impact for an individual could be a positive impact on the wider system.

While we understand the drive to come-up with measurable quantifiable outcomes of recovery innovations [43], there are contradictions inherent to focusing on quantifiable outcomes in relation to mental health recovery. Firstly, the whole philosophy of mental health recovery is that recovery is a journey not an outcome. Also, recovery is individual, and what might be seen as an important outcome to a researcher, a funder, a manager or a government going back to work, or reduced hospital stays, may not be how those with lived-experience of mental health problems would define impact. Furthermore, some outcomes related to organisational culture change may be subtle and difficult to assess because such changes are often complex, slow and multifaceted [44]. For these reasons, we focused our study on impacts rather than outcomes. By asking participants to express what they thought the impacts were in their own words, we were able to conceptualise impacts from the perspective of stakeholders. We believe this framework can inspire other researchers and evaluators to think about impact more broadly when planning studies to investigate impact, outcome or effects of recovery innovations. Like other frameworks in implementation science, it can also be used as a guide to coding data from interviews that explored impact with participants [45, 46].

A number of impacts in our framework are corroborated by existing research across innovation types. In particular, creating opportunities for building or strengthening relationships [9, 21, 22, 38, 47, 48] experiencing personal growth or wellbeing (empowerment, self-confidence (see Additional File 1)) [9, 18–20, 22, 38, 39, 48], and changing mindset [9, 20, 22, 40, 49] were impacts that have been highlighted in previous research for a range of recovery innovations. Some impacts in the categories of *ways of being, ways of thinking* and *ways of interacting* are also reflected in the CHIME framework, a validated conceptual framework for personal recovery [2]. In particular, “creating opportunities for building or strengthening relationships” aligns with “connectedness”, “aspiring to new roles” aligns with “hope and optimism”, and “experiencing personal growth and wellbeing” aligns with “identity”, “meaning”, and “empowerment”. Therefore, our framework for impact does cover the “personal recovery” impacts of recovery innovations, but also goes beyond personal-level impacts which is essential when the focus is on implementing recovery into services and systems.

There are some impacts described in the literature which did not emerge, or did not emerge as explicitly in our analysis. One of those is reducing stigma and self-stigma [19, 20]. We suspect there may be multiple reasons for this. Firstly, the participating organisations in this study were selected based on a thorough examination of readiness for recovery transformation. All the organisations were already committed to implementing recovery and were serving populations with mental health needs. Therefore, it might be that stigmatisation was less prevalent or less overt in these settings, although additional research would be needed to investigate this. Perhaps a more likely explanation is that we, as researchers, never introduced the word stigma in this study in the interview guides. Perhaps, if we had interviewed more service user participants and included questions around stigma, then, participants’ reflections on stigma may have emerged. Two other interrelated impacts of recovery innovations described in the literature are community involvement [42, 47, 48, 50] and increased citizenship [51]. Although we did not label impacts using these terms, we have identified related impacts. For example, “motivating one to aspire to new roles” related to involving oneself in the community (for example the community of residents of a particular building joining committees). The concept of citizenship is closely tied to empowerment [7], and empowerment is well highlighted in our framework both in terms of the impact of the process of being on an implementation team, and of the innovation.

Where we believe our framework makes a unique contribution is in the category *Ways of operating and doing business*. This is because most of the research has so far focused primarily on individual-level impacts of recovery innovations and as a result impacts at the service delivery level have been less well articulated, with the exception being “changing practice” which is well described in other studies [38, 40, 49, 50, 52]. *Shaking things-up* is an impact that demonstrates how recovery transformation involves changing old ways of doing things and disrupting power, and as a result some feathers are likely to be ruffled in the process. Creating a precedent or laying the groundwork is an important impact because system level change often involves innovating structures in the organisation that are not easy to change, and thus doing them once, even if on a small scale, can facilitate future implementation, sustainment, or scaling-up. Inspiring expansion and scaling-up is itself a noteworthy impact, as implementing a recovery innovation is just one element of system transformation and must be embedded in wider change. Implementing a recovery innovation also means that organisations increase their portfolio of services offered to service users and can help empower staff who may have struggled with a lack of options to suggest for service users in the past. As such, service users’ needs are better met, which in the case of this project, was a clear motivator for participating in the study in the first place. Finally, implementing a recovery innovation can reinforce and confirm an organisation’s commitment and push recovery-oriented policies, including guidelines, forward. Implementing guidelines can be challenging as they are broad and involve many actions, raising the question of where to start. Starting with the implementation of one innovation, particularly if the process for doing so empowers organisations to make their own choices, can make important strides in pushing recovery forward into services.

### Strengths, limitations and future research

Version 1 of the IMRI framework is based only on the analysis of empirical data whereas literature reviews are most commonly the basis for conceptual framework development [29, 30]. Our framework will therefore benefit from further refining and validation [29]. For example, by complimenting the empirical data analysis with a literature review and input from an expert panel with experiential and professional expertise, as other authors have done when developing frameworks [2, 18, 19, 38]. A strength of our framework, however, is that it is based on empirical data from multiple sites, implementing multiple recovery innovations, and from multiple stakeholder perspectives being asked about impact at multiple levels. Another direction for future research could be mapping existing quantitative tools for measuring impacts or outcomes in recovery [23, 24, 53] to the IMRI framework, as well as generating a compendium of qualitative interview questions that could explore each impact in future research.

## Conclusions

Recovery-transformation does not happen overnight, but organisations can start with existing guidelines and from them prioritise one recovery innovation that meets their current needs and resources. What our study shows is that even when implementing one recovery innovation, impacts can be wide-reaching, especially when the approach to implementation planning is itself recovery-oriented. We present a first version of the IMRI framework, a conceptual framework to help researchers, evaluators and decision-makers identify and understand impacts grounded in stakeholders’ perspectives.

## Supplementary Information

**Additional file 1.** Concept maps developed in NVivo12 as part of the “interpretation” phase of analysis.

## Data Availability

The datasets used and/or analysed during the current study are available from the corresponding author on reasonable request.
