# Supplementary material for: The impacts of implementing recovery innovations: a conceptual framework grounded in qualitative research
Source: Int J Ment Health Syst. 2022 Oct 9;16:49. doi: 10.1186/s13033-022-00559-2 (PMC9548307; doi:10.1186/s13033-022-00559-2)

Additional file 1

**Concept maps developed in NVivo12 as part of the “interpretation” phase of analysis**

Cite as: Piat et al. The impacts of implementing recovery innovations: A conceptual framework grounded in qualitative research


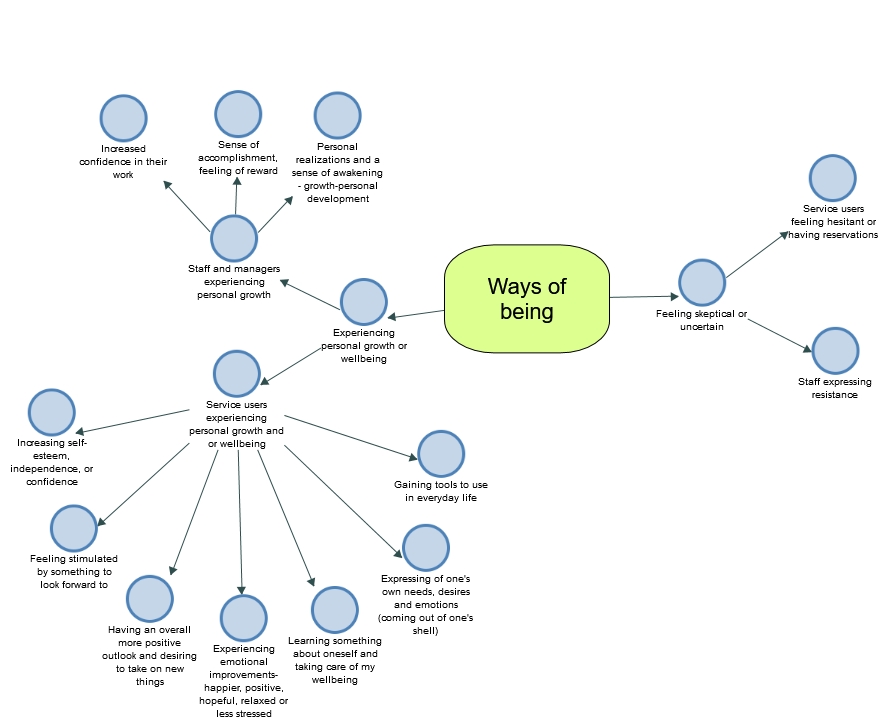


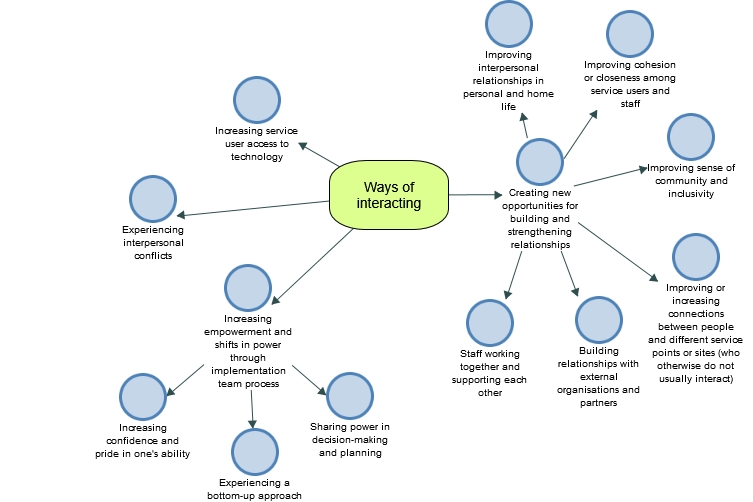


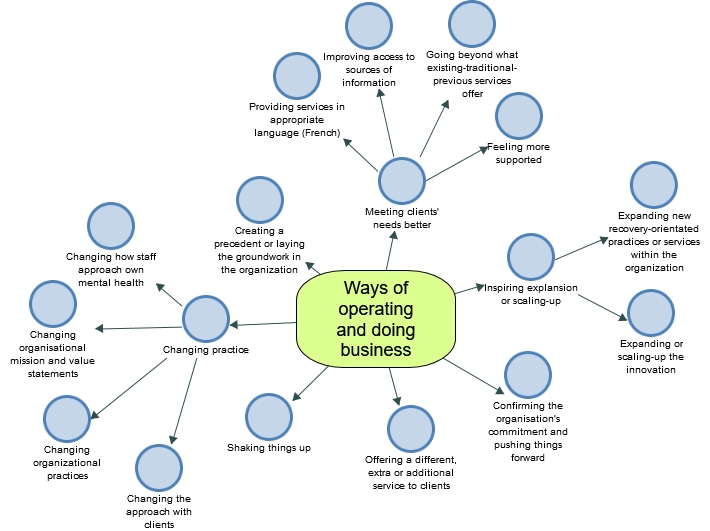


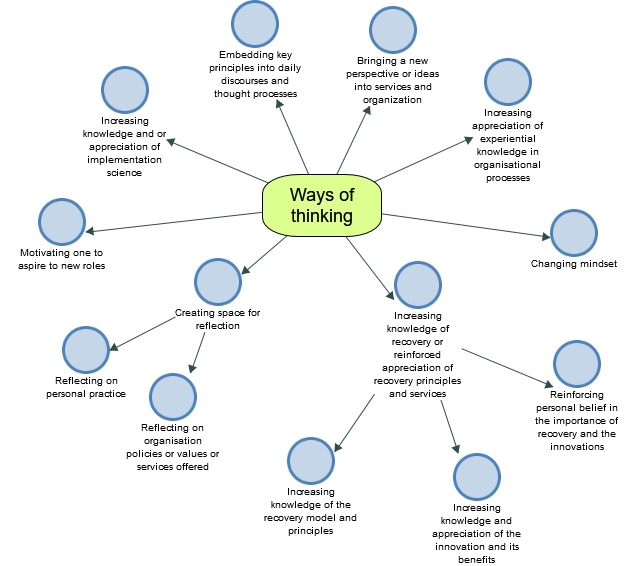

Supplement: Supplementary file 1 — Additional file 1. Concept maps developed in NVivo12 as part of the “interpretation” phase of analysis. [file 13033_2022_559_MOESM1_ESM.docx]
